# Comparative analysis of the repertoire of G protein-coupled receptors of three species of the fungal genus *Trichoderma*

**DOI:** 10.1186/1471-2180-13-108

**Published:** 2013-05-16

**Authors:** Sabine Gruber, Markus Omann, Susanne Zeilinger

**Affiliations:** 1 Research Area Molecular Biotechnology and Microbiology, Institute of Chemical Engineering, Vienna University of Technology, Gumpendorferstrasse 1a, Wien, Austria; 2 current address: Zuckerforschung Tulln GmbH, Josef-Reiter-Strasse 21-23, Tulln, Austria

**Keywords:** Fungi, *Trichoderma*, G protein-coupled receptors, Signaling, Mycoparasitism, Biocontrol

## Abstract

**Background:**

Eukaryotic organisms employ cell surface receptors such as the seven-transmembrane G protein-coupled receptors (GPCRs) as sensors to connect to the environment. GPCRs react to a variety of extracellular cues and are considered to play central roles in the signal transduction in fungi. Several species of the filamentous ascomycete *Trichoderma* are potent mycoparasites, i.e. can attack and parasitize other fungi, which turns them into successful bio-fungicides for the protection of plants against fungal phytopathogens. The identification and characterization of GPCRs will provide insights into how *Trichoderma* communicates with its environment and senses the presence of host fungi.

**Results:**

We mined the recently published genomes of the two mycoparasitic biocontrol agents *Trichoderma atroviride* and *Trichoderma virens* and compared the identified GPCR-like proteins to those of the saprophyte *Trichoderma reesei*. Phylogenetic analyses resulted in 14 classes and revealed differences not only among the three *Trichoderma* species but also between *Trichoderma* and other fungi. The class comprising proteins of the PAQR family was significantly expanded both in *Trichoderma* compared to other fungi as well as in the two mycoparasites compared to *T*. *reesei*. Expression analysis of the PAQR-encoding genes of the three *Trichoderma* species revealed that all except one were actually transcribed. Furthermore, the class of receptors with a DUF300 domain was expanded in *T*. *atroviride*, and *T*. *virens* showed an expansion of PTH11-like receptors compared to *T*. *atroviride* and *T*. *reesei*.

**Conclusions:**

Comparative genome analyses of three *Trichoderma* species revealed a great diversity of putative GPCRs with genus- and species- specific differences. The expansion of certain classes in the mycoparasites *T*. *atroviride* and *T*. *virens* is likely to reflect the capability of these fungi to establish various ecological niches and interactions with other organisms such as fungi and plants. These GPCRs consequently represent interesting candidates for future research on the mechanisms underlying mycoparasitism and biocontrol.

## Background

Fungi are eukaryotes and include organisms with important ecological and economic roles. The relatively simple structure and the ease of cultivation and genetic manipulation make fungi interesting eukaryotic models for studying fundamental biological processes. They share important features with even mammalian cells such as conserved signal transduction pathways that regulate cell function [1,2]; thus studying fungal signaling and environmental sensing contributes to our knowledge on conserved basic molecular principles of life.

Communication of cells with each other and with their environment is crucial for survival of organisms. Consequently, ingenious mechanisms of sensing environmental signals and elaborated ways of adaption to the environment evolved [3]. Cell surface receptors connect the cell to the environment by functioning as sensors. Among these receptors, G protein-coupled receptors (GPCRs) comprise the largest class with roles in virtually every physiological function [4]. GPCRs have a common domain structure containing seven stretches of hydrophobic amino acids spanning the cytoplasmic membrane connected by intra- and extracellular loops with the N-terminus located outside of the cell and the C-terminus within the cytoplasm [5]. The classic paradigm is based on a physical interaction of the GPCR with an intracellular GÎ± subunit once the receptor is activated by ligand binding which leads to dissociation of GÎ± from GÎ²Î³ subunits [6]. Both signalling units then regulate activities of downstream effectors [7-9]. In eukaryotic organisms a plenty of different GPCRs is facing a small amount of G proteins. If G proteins were the only transmitters of GPCR-mediated signaling, this unequal ratio seems to limit the specificity of signal transduction. In recent years several intracellular partners other than G proteins were identified that are capable of mediating signals originating from these receptors. These include arrestins, G protein-coupled receptor kinases, small GTP-binding proteins, and many more [10-13]. Accordingly, GPCRs are extremely diverse in sequence and function and missing genome sequence information and constraints in structure prediction for a long time impaired research on these proteins. Although pheromone- and nutrient- sensing GPCRs have been studied extensively in yeast and some filamentous fungi [14-26] far more GPCRs remain to be identified and characterized.

The fungal genus *Trichoderma* comprises saprophytic and mycoparasitic species, and species interacting with plants and animals [27]. Because of these versatile lifestyles and the variety of interactions with other organisms, *Trichoderma* fungi are valuable models for studying organismic cross-talk and signaling. Studies on heterotrimeric G proteins revealed a multitude of processes being regulated by these signal transduction compounds in *Trichoderma*. The class I adenylate cyclase-inhibiting as well as the class III adenylate cyclase-activating GÎ± subunits regulate vegetative growth and conidiation of the fungus and affect processes relevant for mycoparasitism [28], i.e. a lifestyle where *Trichoderma* parasitizes other fungi. *Trichoderma atroviride* Tga1 as well as Tga3 govern the production of extracellular chitinases and antifungal metabolites, and Tga3 is essential for transmitting signals that regulate the recognition of the host fungus and attachment to its hyphae. Both, *T*. *atroviride* âˆ†*tga1* as well as âˆ†*tga3* mutants, are unable to overgrow and lyse host fungi [29-31], while *Trichoderma virens* TgaA regulates mycoparasitism in a host-specific manner [32]. For *T*. *virens* âˆ†*tgaB* mutants missing the class II GÎ±-encoding gene, unaltered growth, conidiation, and mycoparasitic activity have been reported [32]. In the saprophyte *Trichoderma reesei*, the heterotrimeric G protein pathway is crucial for the interconnection of nutrient signaling and light response. Besides the GÎ± subunits GNA1 and GNA3, which transmit signals positively impacting cellulase gene expression, GNB1 (GÎ²), GNG1 (GÎ³) and the phosducin PhLP1 influence light responsiveness, glycoside hydrolase expression and sexual development [33,34].

Here we present an exploration of the genomes of the two mycoparasites *T*. *atroviride* and *T*. *virens* and identify members of the G protein-coupled receptor family from the entire deduced proteomes. The identified proteins are classified and compared to those encoded in the saprophyte *T*. *reesei* and several other fungi. In contrast to the presence of only three GÎ± subunits, one beta and one gamma subunit in each of the genomes of the three *Trichoderma* species, our analyses revealed a great diversity of GPCRs and differences both between the three *Trichoderma* species and between *Trichoderma* and other fungi.

## Results and discussion

### *Identification of G protein*-*coupled receptor*-*like proteins in the genomes of three* Trichoderma *species*

The *T*. *atroviride*, *T*. *virens* and *T*. *reesei* genome databases were searched for putative GPCRs using a homology (BLAST)-based strategy. Together with the putative GPCRs identified in the genome of *Neurospora crassa*[2] and *Phytophtora sojae* GPR11 [35], the 18 GPCRs previously identified in *Aspergillus* spp. [1] and the three new GPCRs predicted in the *Verticillium* genome [36] were used in a BLASTP search against the predicted proteomes of the following species of the Sordariomycetes (*Magnaporthe grisea*, *Podospora anserina*, *Chaetomium globosum*, *Fusarium graminearum*, *Nectria haematococca*, *T*. *reesei*, *T*. *atroviride* and *T*. *virens*), a subgroup within the Ascomycota. In an analogous manner, the PTH11 receptor of *M*. *grisea*[14,37] was used as a query. All consequently identified GPCR-like proteins were next used as a query in similar BLAST searches of the proteomes of the other species. In the end each possible combination was tested. By additionally applying a HMM-based approach, which is suitable for detecting candidates lacking significant sequence similarity to known GPCR-like proteins and therefore may escape detection by BLAST-based homology searches, two additional proteins of the PTH11-like class could be identified (Triat86665, Trive78137).

All identified *Trichoderma* proteins were evaluated for the typical topology of seven transmembrane regions and, if conducive, a manual editing of candidate GPCR sequences was performed including movement of exon-intron boundaries and sequence extension or truncation. This total set of analyses resulted in the identification of 65 and 76 putative GPCRs in *T*. *atroviride* and *T*. *virens*, including 38 and 52 PTH11-like receptors, respectively, which are facing 58 predicted GPCRs in the *T*. *reesei* genome (Table 1). Among the PTH11-like receptors, a protein exhibiting 15 transmembrane domains was found in all three *Trichoderma* species. An orthologue of this putative GPCR has previously been identified in *M*. *grisea* and *A*. *nidulans*[2] suggesting conservation of this particular receptor.

**Table 1:** **Classification of putative GPCRs identified in the genomes of*T***. ***atroviride***, ***T***. ***virens***, **and*T***. ***reesei***

**GPCR class**	***T.atroviride***	***T.virens***	***T.reesei***	**Characteristics/domains**
I (pheromone receptors)	ID 36032	ID 147400	ID 64018 (HPR1)	STE2-type
II (pheromone receptors)	ID 147894	ID 40681	ID 57526 (HPR2)	STE3-type
III (related to *A*. *nidulans* GprC, GprD, and GprE)	ID 246916	ID 29548	ID 59778	Git3 (G protein-coupled glucose receptor) domain
IV (nitrogen sensors)	ID 238619	ID 41902	ID 80125	PQ-loops
	ID 300620	ID 83179	ID 4508	
V (cAMP receptor-like)	ID 160995 (Gpr1)	ID 33049	ID 123806	Secretin-family/ Dicty_CAR domain
	ID 50902 (Gpr2)	ID 51368	ID 72004	
	ID 83166	ID 67397	ID 72627	
	ID 81233	ID 57873	ID 72605	
VI (GPCRs containing RGS domain)	ID 293686	ID 45779	ID 63981	RGS-domain
	ID 40423	ID 78031	ID 81383	
	ID 210761	ID 40202	ID 37525	
VII (related to rat growth hormone releasing factor)	ID 133045	ID 146164	ID 53238	Secretin-like
VIII (related to human steroid receptor mPR)	ID 290047	ID 30459	ID 119819	HlyIII-superfamily
	ID 210209	ID 47976	ID 68212	
	ID 142946	ID 160502	ID 70139	
	ID 46847			
	ID 152366	ID 194061		
	ID 142943	ID 92622	ID 82246	
	ID 136196	ID 180426	ID 56426	
IX (microbial opsins)	ID 210598	0	0	Bac_rhodopsin
X (similar to PTM1)	ID 210445	ID 90826	ID 5979	Lung_7TM superfamily
XI (similar to GPCR89)	ID 93659	ID 160103	ID 107503	ABA_GPCR domain
XII (family C-like GPCRs)	ID 130836	ID 179509	ID 55374	
XIII (related to GPR11 of *P*. *sojae*)	ID 136442	ID 13017	ID 120238	DUF300 superfamily
	ID 152316	ID 15638	ID 27948	
	ID 296436			
PTH11-like	38 members	52 members	35 members	related to *M*. *grisea* PTH11 receptor

**Figure 1:**
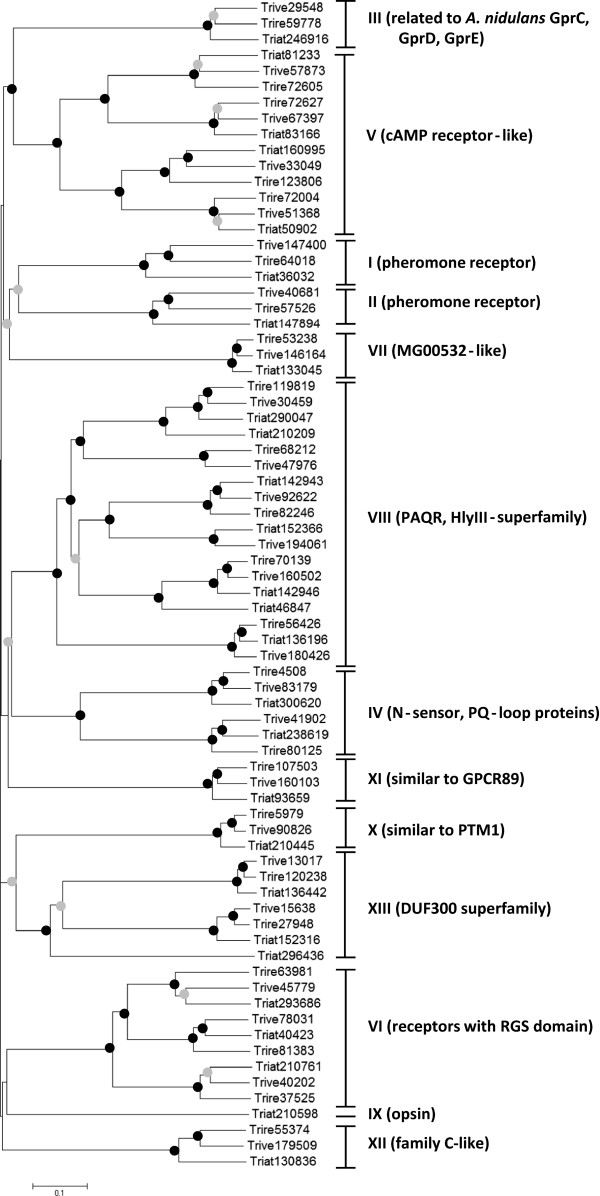
**Phylogenetic analysis of predicted GPCRs(except PTH11-like proteins)identified in the genomes of the two mycoparasites*T.atroviride*and*T.virens,*and the saprophyte*T.reesei*.** The 7TM regions were aligned and the tree was constructed using neighbor-joining methods resulting in a grouping into 13 classes (I-XIII). Classes were numbered according to former classification schemes [12,36]. Nodes supported with bootstrap values above 70% (1000 repetitions) are indicated with a black dot, nodes with bootstrap values between 50 -70% are indicated with a grey dot, bootstrap values less than 50% were removed.

Proteins were grouped into classes according to phylogenetic analyses (Figure 1, Additional file 1). A list of PTH11-like GPCRs is given in Additional file 2.

### *Phylogenetic analysis of the identified* Trichoderma *GPCR*-*like proteins*

Previous studies led to the categorization of fungal GPCRs into the following classes: pheromone receptors, carbon sensors, putative nitrogen sensors, cAMP receptor-like proteins, GPCRs with an RGS domain, GPCRs related to rat growth hormone releasing factor, mPR-like GPCRs, microbial opsins and those related to *M*. *grisea* PTH11 [1,2,14]. Recently, this classification has been extended by three novel classes whose members show similarity to PTM proteins (putative tumor necrosis factor receptors), to GPR89A of higher eukaryotes, and to family C-like GPCRs (metabotropic glutamate/pheromone receptors of *Gallus gallus*), respectively [36].

A phylogenetic analysis of all putative GPCRs identified in this study including those previously described for *T*. *reesei*[38,39] revealed that the *Trichoderma* proteins were distributed over 14 classes including PTH11-like GPCRs and putative receptors similar to *P*. *sojae* GPR11 (Figure 1, Table 1). Phylogeny also showed that the orthologous proteins from *T*. *atroviride*, *T*. *virens* and *T*. *reesei* mainly formed the topologies ((Tr, Tv) Ta) and ((Ta, Tv) Tr) with 14 and 9 cases, respectively, whereas the ((Ta, Tr) Tv) topology resulted only once (Figure 1). This suggests that some of the GPCRs of *T*. *virens* are more related to those of *T*. *atroviride* and some are more related to those of *T*. *reesei*. This is in accordance to the phylogeny of these species based on other genes showing that *T*. *atroviride* resembles the more ancient state of *Trichoderma* and that both *T*. *virens* and *T*. *reesei* evolved later [40]. Accordingly, comparative genome analysis showed that the lineage to *T*. *reesei* appears to have lost a significant number of genes present in *T*. *atroviride* and maintained in *T*. *virens*[40].

### Trichoderma *members of classes I to VII of fungal GPCRs*

Two putative pheromone receptors are encoded in the genomes of the three *Trichoderma* species analyzed. Similar to other fungi, these proteins group to classes I and II of fungal GPCRs (Figure 1, Additional file 1), respectively, and harbor the typical STE2 (pfam02116; Triat36032, Trive147400, Trire64018) and STE3 (pfam02076; Triat147894, Trive40681, Trire57526) domains. Functional analysis of the pheromone receptors of *T*. *reesei* (*H*. *jecorina*) showed that HPR1 and HPR2 confer female fertility in their cognate mating types, mediate induction of fruiting body development, and are involved in ascosporogenesis [23]. While sexual crossing remains to be experimentally shown for *T*. *atroviride* and *T*. *virens*, a respective *MAT1*-*2* mating type locus is present in their genomes and the corresponding teleomorphs, *Hypocrea atroviridis* and *Hypocrea virens*, have already been described [41,42].

There is no direct sequence homologue of the class III carbon-sensing GPCRs Gpr1 of *Saccharomyces cerevisiae* and GPR-4 of *N*. *crassa*[21,43,44] in *Trichoderma*. Nevertheless, we could identify a 7-transmembrane domain protein in *T*. *atroviride* (Triat246916), *T*. *virens* (Trive29548) and *T*. *reesei* (Trire59778) sharing sequence and structural similarity with *Aspergillus nidulans* GprC, GprD and GprE, and GprC and GprD of *Aspergillus fumigatus* and *Aspergillus oryzae*, which have previously been described as class III GPCRs [1]. GprD negatively regulates sexual development in *A*. *nidulans* and *A*. *fumigatus* and GprC and GprD of *A*. *fumigatus* are furthermore involved in integrating and processing stress signals via modulation of the calcineurin pathway [45,46]. Recently, GprD was further shown to be involved in the sensing of oxylipins in *A*. *nidulans* and *A*. *flavus*[47]. Due to the absence of a locus similar to that of *N*. *crassa* GPR-4 in the *T*. *reesei* genome, it has been postulated that *T*. *reesei* does not possess a class III GPCR. Trire59778 was instead grouped to the cAMP receptor-like class [39]. However, structural analyses of receptors of classes III and V revealed distinct topologies: whereas class III members display seven transmembrane regions at their amino-terminal end and a long carboxy-terminal cytoplasmic domain, class V receptors exhibit five domains at the N-terminal end, a long intracellular loop and two helices next to the C-terminus [1]. Consistent with a clustering of Triat246916, Trive29548 and Trire59778 with *A*. *nidulans* GprC, GprD and GprE in the phylogenetic analysis (Additional file 1), the *Trichoderma* proteins clearly share the topology of class III members and contain a Git3 (pfam11710; G protein-coupled glucose receptor) domain. Whether these proteins actually are implicated in glucose sensing, remains to be elucidated.

Fungal GPCRs with similarity to *Schizzosaccharomyces pombe* Stm1 have been designated as class IV. The Stm1 receptor has been previously shown to be required for proper recognition of nitrogen starvation signals and to couple to the Gpa2 GÎ± subunit in *S*. *pombe*[48]. This class of GPCRs, all containing PQ-loop repeats, is well conserved in filamentous fungi [2], although their function remains elusive. Two PQ-loop containing 7-transmembrane proteins grouping to class IV are encoded in the mycoparasites *T*. *atroviride* and *T*. *virens* (Figure 1, Table 1) which is consistent with previous reports on *T*. *reesei*[38,39]. Interestingly, one of the two class IV members of *T*. *atroviride*, Triat300620, has been found in an EST-based study to be expressed exclusively under mycoparasitic conditions (i.e. in direct confrontation with the host fungus *Rhizoctonia solani*) [49]. This transcriptome analysis further revealed that *T*. *atroviride* faces stress from nitrogen limitation when it is confronted with a fungal host accompanied by an up-regulation of genes encoding proteolytic enzymes. Consequently, oligopeptides emerging from an initial degradation of the host by secreted proteases have been suggested as signals for nitrogen deficiency by binding to the Stm1-receptor in a ligand-receptor-specific manner [49]. A possible role of Triat300620 in nitrogen signaling during mycoparasitism is further supported by the fact that *T*. *atroviride* knock-out mutants missing the Tga3 GÎ± protein (orthologue of *S*. *pombe* Gpa2) are completely deficient in mycoparasitism, e.g. unable to attack and parasitize host fungi [31].

The class V of fungal GPCRs comprises cAMP receptor-like (CRL) proteins that are distantly related to the four cAMP receptors of *Dictyostelium discoideum*[1,2]. Similar to *T*. *reesei*[38], four CRL proteins harboring a Dicty_CAR (pfam05462) domain were identified in the genomes of the two mycoparasitic *Trichoderma* species *T*. *atroviride* and *T*. *virens* (Figure 1, Table 1). Two of these (Gpr1/ Triat160995 and Gpr2/ Triat 50902) have been functionally characterized in *T*. *atroviride*. While mutants silenced in the *gpr2* gene did not show any phenotypic alterations [28,38], *gpr1* mutants were unable to attach to host hyphae and to respond to host fungi with the production of cell wall-degrading enzymes. Besides these defects in mycoparasitism-relevant activities, Gpr1 further affects vegetative growth and conidiation of *T*. *atroviride*[50]. As Gpr1 did not interact with any of the three *T*. *atroviride* GÎ± proteins (Tga1, Tga2, or Tga3) in a split-ubiquitin yeast-two-hybrid assay [50], signal transduction in a G protein-independent manner cannot be ruled out at the moment.

Members of class VI of fungal GPCRs are characterized by the presence of both 7-transmembrane regions and an RGS (regulator of G protein signaling) domain in the cytoplasmic part of the proteins. They show similarity to *Arabidopsis thaliana* AtRGS1 which modulates plant cell proliferation via the Gpa1 GÎ± subunit [51]. In contrast to other filamentous ascomycetes like *F*. *graminearum*, *N*. *crassa*, *A*. *nidulans*, *A*. *fumigatus*, *A*. *oryzae*, *Verticillium* spp. and *M*. *grisea*, which possess only one or two members of class VI [1,2], three putative RGS domain-containing GPCRs could be identified in both *T*. *reesei*[38,39] and the two mycoparasitic species *T*. *atroviride* and *T*. *virens* (Table 1).

A putative receptor distantly related to mammalian GPCRs like the rat growth hormone-releasing factor receptor has been initially identified in the *M*. *grisea* genome [14]. Similar to closely related fungi like *N*. *crassa* and *F*. *graminearum* one orthologue with more than 50% amino acid identity to MG00532 is encoded in the genomes of *T*. *atroviride*, *T*. *virens* and *T*. *reesei* which accordingly was assigned to class VII (Table 1).

### *The PAQR family is expanded in mycoparasitic* Trichoderma *species*

Receptors responding to progesterone and adiponectin as ligands have previously been classified as progestin-adipoQ receptors (PAQR [52], a group of 7-transmembrane proteins lacking significant sequence similarity to any previously described GPCRs but with ancient evolutionary roots. The PAQR family also includes prokaryotic hemolysin-type proteins and members have been identified throughout the eukaryotic kingdom including 11 paralogues in mammals [52]. In *S*. *cerevisiae* the PAQR family members Izh1p, Izh2p, Izh3p, and Izh4p are involved in the regulation of intracellular zinc levels. Izh2p has further been reported to play a role in lipid and phosphate metabolism [53,54], and to function as a receptor for the plant defense protein osmotin which induces programmed cell death in yeast [55].

**Figure 2:**
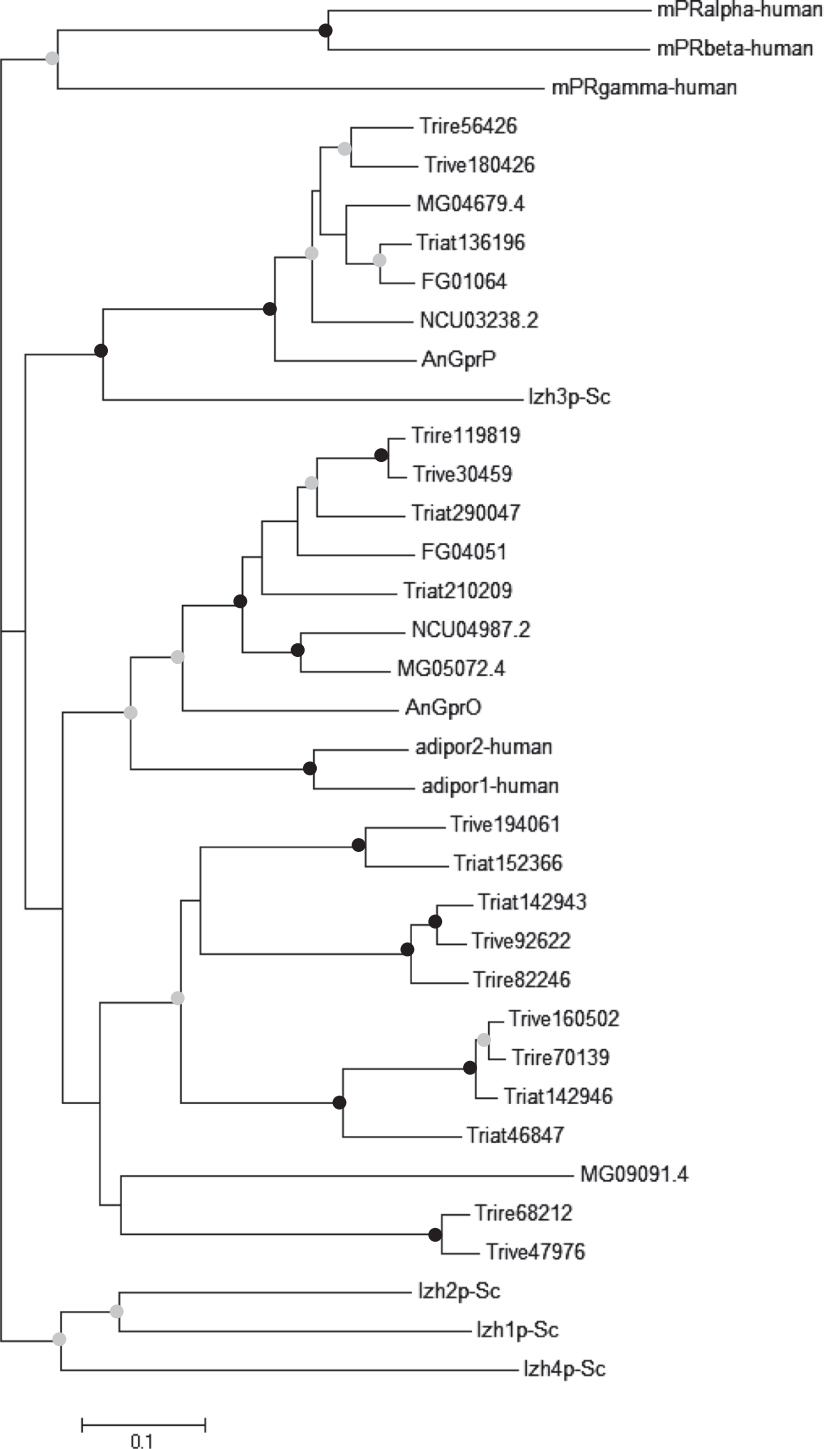
**Phylogenetic analysis of PAQR family(class VIII)members.** PAQR members identified in the genomes of the three *Trichoderma* species and those present in *N*. *crassa* (NCU03238, NCU04987), *A*. *nidulans* (AnGprP, AnGprO), *F*. *graminearum* (FG04051, FG01064), *M*. *grisea* (MG0901, MG05072, MG04679), *S*. *cerevisiae* (Izh1p, Izh2p, Izh3p, Izh4p), and the human mPR (mPR-alpha, -beta, -gamma) and adiponectin-receptors (adipor1, adipor2) were aligned using ClustalX. The alignment was then processed using the Gblocks server [56] and the tree was constructed using neighbor-joining methods. Nodes supported with bootstrap values above 70% (1000 repetitions) are indicated with a black dot, nodes with bootstrap values between 50 -70% are indicated with a grey dot, bootstrap values less than 50% were removed.

In the genomes of filamentous fungi such as *N*. *crassa*, *A*. *nidulans*, *F*. *graminearum*, and *M*. *grisea* two to three PAQR-type proteins are encoded and have been designated as class VIII of fungal GPCRs [1,2]. Our mining of the genomes of *T*. *virens* and *T*. *atroviride* revealed the presence of six and seven PAQR members (Table 1, Figure 1), respectively, all of which bear the hemolysin III motif (pfam03006, HlyIII) and which face five members identified in *T*. *reesei*[38,39]. Phylogenetic analysis showed the *Trichoderma* orthologues Triat136196, Trive180426, Trire56426 in a clade together with yeast Izh3 (Figure 2). Izh3 possesses a long N-terminal tail with unknown function as a distinctive characteristic [55]. Similar extracellular N-terminal extensions of ~280 amino acids were found in the *Trichoderma* Izh3-like proteins Triat136196, Trive180426 and Trire56426. It is worth mentioning that some of the *Trichoderma* class VIII members do not share the typical GPCR topology but have an extracellular C-terminus and the N-terminal domain within the cytoplasm. Triat210209, Triat46847, Triat142943, Trire82246, Trive92622 are in the same, although not well supported, cluster with the human adinopectin receptors adipor1-human and adipor2-human, which share the same topology [52].

**Figure 3:**
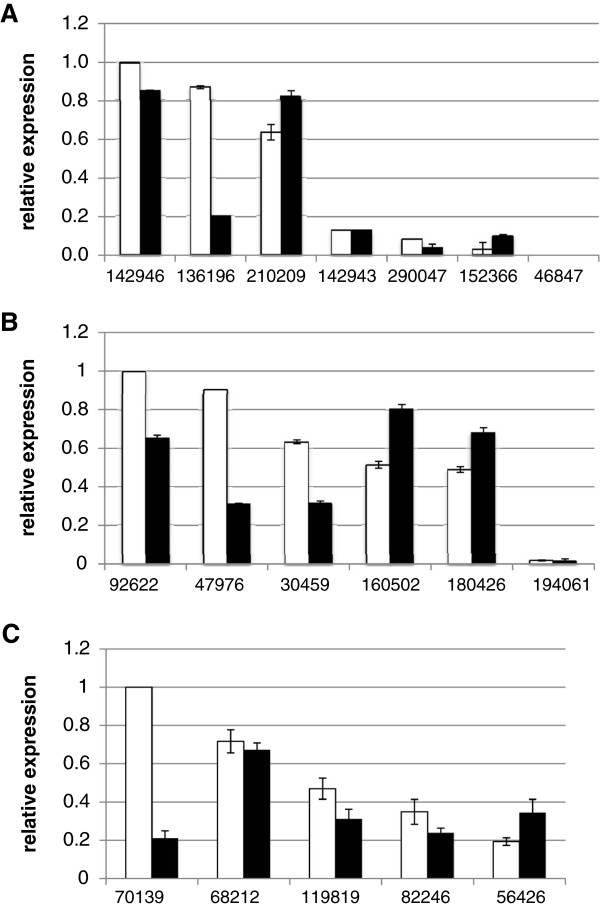
**Relative transcription ratios of PAQR family(class VIII)members.** mRNA levels of the respective genes of *T*. *atroviride* (**A**), *T*. *virens* (**B**) and *T*. *reesei* (**C**) upon direct contact with the host fungus *R*. *solani* (black bars) were assessed by RT-qPCR and compared to a control where the respective *Trichoderma* species was grown alone (white bars). Samples of the gene with highest expression in the control condition were arbitrarily assigned the factor 1. *sar1* was used as reference gene.

To analyze whether the class VIII genes identified in the *Trichoderma* genomes are actually transcribed, their expression was assessed by RT-qPCR. Respective transcripts were detected for all five and six genes of *T*. *reesei* and *T*. *virens*, respectively, as well as for six of the seven genes identified in the *T*. *atroviride* genome (Figure 3). Triat46847 was not transcribed under the growth condition tested (PDA). Analysis of mRNA levels after co-cultivation of *Trichoderma* with *Rhizoctonia solani* revealed a significantly enhanced expression of Trive160502 (pâ€‰=â€‰0.000) and Trive180426 (pâ€‰=â€‰0.031) in *T*. *virens*, Triat152366 (pâ€‰=â€‰0.027) and Triat210209 (pâ€‰=â€‰0.000) in *T*. *atroviride*, and Trire56426 (pâ€‰=â€‰0.000) in *T*. *reesei* upon contact with the host fungus (Figure 3). On the other hand, expression of Triat142946 (pâ€‰=â€‰0.000), Triat136196 (pâ€‰=â€‰0.000) in *T*. *atroviride*, Trive92622 (pâ€‰=â€‰0.000), Trive47976 (pâ€‰=â€‰0.000), Trive30459 (pâ€‰=â€‰0.034) in *T*. *virens*, and Trire70139 (pâ€‰=â€‰0.032), Trire119819 (pâ€‰=â€‰0.000) in *T*. *reesei* was significantly decreased in the presence of *R*. *solani* compared to the corresponding controls. Transcript levels of Triat290043 (pâ€‰=â€‰0.971), Triat142943 (pâ€‰=â€‰0.093), and Trire82246 (pâ€‰=â€‰0.102) were unaffected by the presence of *R*. *solani*. Again no transcript could be detected for Triat46847. Expression of Triat46847 was further assessed on both plates and in liquid minimal and full media and under different developmental stages (vegetative growth, conidiation) of the fungus. No transcript could be detected under all the conditions tested (data not shown).

**Figure 4:**
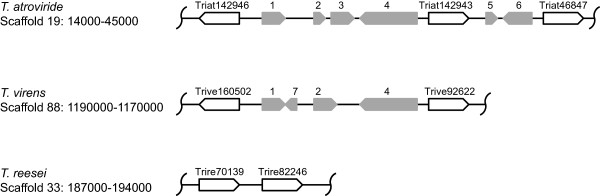
**Schematic drawing of the*T.atroviride*genomic locus with the PAQR(class VIII)-encoding genes Triat142946, Triat142943, and Triat46847 and the loci with their orthologues in*T.virens*and*T.reesei*.** Scaffolds and position numbers are given as specified in the respective genome databases [57-59]. PAQR-encoding genes are indicated by white arrows; other genes are given in grey (1: Triat47305/Trive123162, putative subtilisin-like peptidase; 2: Triat178339/Trive160495, putative ankyrin repeat domain protein; 3: Triat255480, putative ankyrin repeat domain protein; 4: Triat215171/Trive160757, hypothetical NACHT and ankyrin domain protein; 5: Triat305654, predicted small secreted cystein-rich protein; 6: Triat290393, hypothetical protein; 7: Trive66658, hypothetical protein).

Analysis of the location of the seven PAQR-encoding genes in the genome of *T*. *atroviride* revealed that three of them (Triat142946, Triat142943, Triat46847) are in close vicinity on scaffold 19 (Figure 4). This is similar in *T*. *virens* and *T*. *reesei* for the orthologues of Triat142946 and Triat142943 suggesting the possibility that the third *T*. *atroviride* gene (Triat46847), which was found not to be expressed under any of the conditions tested, may have resulted from gene duplication with subsequent inactivation.

The finding that the genes located in the genomes of both *T*. *atroviride* and *T*. *virens* between the orthologous receptor triplets Triat142946/Trive160502/Trire70139 and Triat142943/Trive92622/Trire82246 have been lost in *T*. *reesei* (Figure 4) is consistent with a reported paralogous gene expansion in *T*. *atroviride* and *T*. *virens* compared to *T*. *reesei* and other non-mycoparasitic fungi [40].

After the class of PTH11-like receptors, the PAQR family is the second largest GPCR class in *Trichdoderma*. The expansion of the PAQR family especially in *T*. *atroviride* and *T*. *virens* together with the fact that *S*. *cerevisiae* Izh2 was found to regulate fungal development in response to plant osmotin [55], make these receptors interesting candidates for an involvement in interspecies communication between *Trichoderma* and other (host) fungi and/or plants. The importance of fungal class VIII GPCRs in environmental sensing is further supported by the recent characterization of a PAQR family member of the fungus *Sporothrix schenkii*. SsPAQR1 was found to respond to the steroid hormone progesterone by signaling via the GÎ± subunit SSG-2 [60].

### Trichoderma *members of classes IX to XII of fungal GPCRs*

A 7-transmembrane protein with a bacteriorhodopsin domain is encoded in the genome of *T*. *atroviride*. Triat210598 is orthologous to *N*. *crassa* NOP-1 and ORP-1 and *A*. *nidulans* NopA (Additional file 1). Interestingly, Triat210598 has no homologs in *T*. *reesei* and *T*. *virens*. Due to the finding that Triat210598 is located in a non-syntenic genome region it has been suggested that *T*. *reesei* and *T*. *virens* have lost this gene during evolution [33]. This hypothesis is in agreement with recent results showing that *T*. *reesei* and *T*. *virens* are derived relative to *T*. *atroviride*, the latter resembling the more ancient state of *Trichoderma*[40].

Classes X, XI, and XII of fungal GPCRs have recently been defined in *Verticillium* spp. [36]. Similar to *Verticillium* and other filamentous fungi such as *A*. *nidulans*, *M*. *grisea*, *N*. *crassa*, and *F*. *graminearum*, one putative PTM1-like GPCR was identified in the two mycoparasites *T*. *atroviride* and *T*. *virens* as well as the saprophyte *T*. *reesei*. Consistent with the presence of a Lung_7-TM_R domain (pfam06814) and similarity to the putative tumor necrosis factor receptor-like GPCR PTM1 of *S*. *cerevisiae*, the respective *Trichoderma* proteins were designated as class X members (Table 1).

One putative member related to human GPR89A was identified in the genome of each of the three *Trichoderma* species (Table 1). The *Trichoderma* proteins showed the typical structure previously described for receptors of class XI with 9 transmembrane regions and a large third cytoplasmic loop [36], and contain a ABA_GPCR (pfam12430; abscisic acid G protein-coupled receptor) domain.

Putative fungal receptors with similarity to family C-like GPCRs (metabotropic glutamate/pheromone receptors) have previously been defined as class XII [36]. Similar to other filamentous ascomycetes, one putative GPCR grouping to this class was identified in each of the three *Trichoderma* species. Whereas the respective proteins of both *T*. *atroviride* and *T*. *reesei* exhibit the typical structure with 7 transmembrane domains and the long C-terminal tail, the *T*. *virens* homologue (Trive179509) only exhibits 6 transmembrane regions.

### *PTH11*-*Related proteins of* Trichoderma

The PTH11 receptor was first identified in *M*. *grisea*, where it is required for host surface recognition and pathogenicity [37]. PTH11 has an extracellular amino-terminal CFEM domain followed by seven transmembrane regions and PTH11-related proteins are restricted to fungi belonging to the subphylum Pezizomycotina [14].

**Figure 5:**
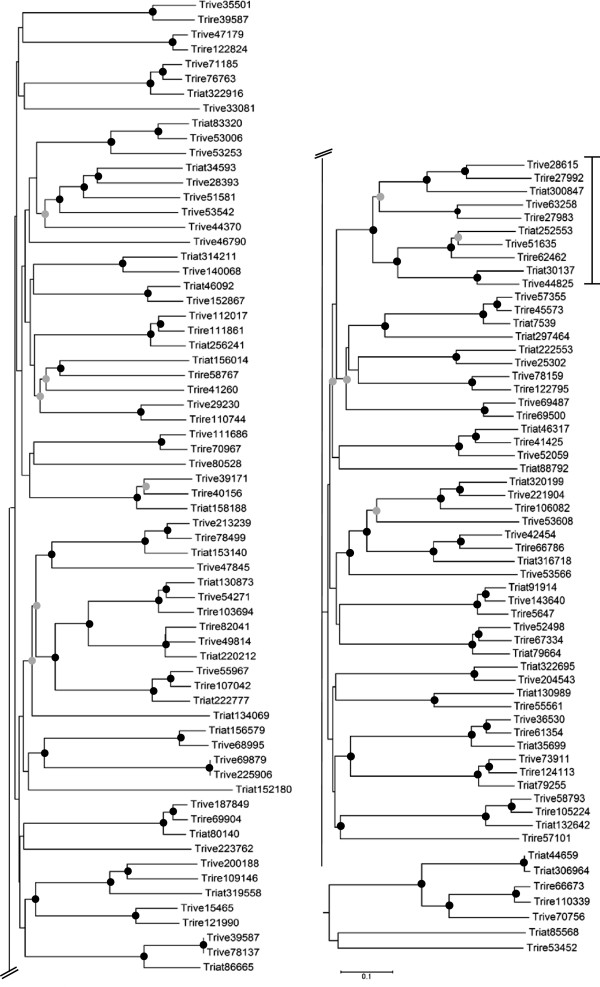
**Neighbor**-**joining tree of PTH11**-**related proteins identified in the genomes of the three*Trichoderma*species.** The clade containing proteins with a CFEM domain is marked with a black line. Nodes supported with bootstrap values above 70% (1000 repetitions) are indicated with a black dot, nodes with bootstrap values between 50 -70% are indicated with a grey dot, bootstrap values less than 50% were removed.

In both the mycoparasitic *Trichoderma* species as well as *T*. *reesei*[38,39], the number of identified PTH11-like proteins was higher than in the saprophyte *N*. *crassa* (25 members) but lower than in the plant pathogens *M*. *grisea* (61 members) and *F*. *graminearum* (106 members) [2,14]. Similar to the above mentioned fungi, only a subset of the identified *Trichoderma* proteins contained the fungal-specific cysteine-rich CFEM (pfam05730) domain (Figure 5, Additional file 2), which is characteristically present in the extracellular region of some membrane proteins with proposed roles in fungal pathogenicity. Compared to *T*. *atroviride* (38 members) and *T*. *reesei* (35 members), we found a marked expansion of PTH11-related proteins in *T*. *virens* (52 members).

### *Additional putative GPCRs of* Trichoderma *which are beyond the existing classification system of fungal GPCRs* (*class XIII*)

Recently, a putative GPCR of *Phytophtora sojae* (GPR11) controlling zoospore development and virulence of *P*. *sojae* to soybean has been described [35]. Performing a BLASTP search with GPR11 as a query against the proteomes of *T*. *atroviride*, *T*. *virens*, *T*. *reesei*, and those of *N*. *crassa*, *M*. *grisea*, and *A*. *fumigatus* revealed respective orthologues in all fungi tested. Whereas in *T*. *atroviride* three proteins were identified (Table 1), *T*. *reesei* and *T*. *virens* as well as the other ascomycetes possess two members each. All putative *Trichoderma* GPCRs identified this way have a DUF300 domain (domain of unknown function, pfam03619). Such a domain is also present in e.g. the class A GPCRs Cand9 and Cand10 of *Arabidopsis thaliana*[61] and *P*. *sojae* GPR11. Topological analysis of the *Trichoderma* proteins revealed a heptahelical topology with three N-terminal transmembrane regions, a long second cytoplasmic loop followed by four transmembrane regions and a long intracellular loop at the C-terminus. As these putative GPCRs represented a separate clade in the phylogenetic analysis (Figure 1), they were assigned to a new class (class XIII, Table 1) thereby extending the classification system of fungal GPCRs to 14 classes.

## Conclusions

A thorough examination of the genomes of the two mycoparasites *T*. *atroviride* and *T*. *virens* and the saprophyte *T*. *reesei* for putative GPCRs revealed for most classes a high conservation of their number and structure within this genus. On the other hand, remarkable differences in individual classes were found among the three *Trichoderma* species and among *Trichoderma* and other filamentous fungi. Whereas for class I to VII members, orthologous triplets with similar length and sequence are present in the genomes of the three *Trichoderma* species and their number is also similar to other fungi, the PAQR family has expanded especially in *T*. *atroviride*. Considering the identification of members of classes X, XI, and XII and proteins similar to the *P*. *sojae* GPR11 receptor in *Trichoderma*, the presented 14 classes now define the most comprehensive classification system for GPCR-like proteins of fungi. The huge diversity of GPCRs in *Trichoderma* spp. and especially in the mycoparasites is likely to reflect the capability of these fungi to establish various ecological niches and interactions with other organisms.

It is worth mentioning that with the exception of few members, the proteins identified as putative GPCRs in this study have only been characterized *in silico*. Taking into account that only three Î±, one Î² and one Î³ subunit of heterotrimeric G proteins are encoded in the *Trichoderma* genomes which face more than 55 GPCRs, studying the signaling output and identifying the respective intracellular interaction partners of those receptors will provide interesting insights on how these fungi adapt to their different lifestyles.

## Methods

### *Identification of GPCR*-*encoding genes of* Trichoderma atroviride *and* Trichoderma virens

Version 2 of the *T*. *atroviride* genome database [57] comprises 11,863 gene models on 29 scaffolds; version 2 of the *T*. *virens* genomic sequence [58] comprises 12,427 gene models on 93 scaffolds. For the homology-based search of GPCR-like proteins from *T*. *atroviride* and *T*. *virens*, the genomic sequences and deduced proteomes of the following fungi were used: *Trichoderma reesei*[59]*Aspergillus nidulans*, *Aspergillus fumigatus*, *Aspergillus oryzae*[62], *Neurospora crassa*[63], *Magnaporthe grisea*[64], *Podospora anserine*[65], *Chaetomium globosum*[66], *Fusarium graminearum*[67], and *Nectria haematococca*[68]. An e-value limit of 1e-09 was applied.

To identify putative GPCRs within the *T*. *atroviride* and *T*. *virens* proteomes that lack significant sequence similarity to known GPCR-like proteins and therefore may escape detection by homology search, a more sensitive database searching using hidden Markov models (HMM) was performed using the program HMMER (http://hmmer.janelia.org/) [69].

All obtained predicted proteins were analyzed with the TMHMM, ConPred II and HMMTOP algorithms [70-72] to test for the typical 7-transmembrane domain topology. For those few proteins exhibiting less than seven transmembrane domains, the respective encoding gene and flanking regions were retrieved from the genome database and examined manually. Wrongly predicted intron-exon boundaries were mainly found and manually corrected resulting in the detection of the missing transmembrane domains.

### Protein alignments and phylogenetic analysis

The classification system of Lafon et *al*. [1], which classifies fungal GPCRs into nine classes according to their sequence similarity, was applied to all detected putative GPCRs of *Trichoderma*. In addition, members of the three additional classes identified in *Verticillium* spp. [36], and the GPR11 protein of *Phytophtora sojae*[35] were used to identify and classify respective members of *T*. *atroviride*, *T*. *virens* and *T*. *reesei*. Multiple sequence alignments of the identified putative GPCR-like proteins and phylogenetic trees with a neighbor-joining approach were generated using ClustalX [73]. A bootstrap with 1000 repetitions was included.

### Cultivations and RT-qPCR analysis

*T*. *atroviride* strain P1 (ATCC 74058; teleomorph *Hypocrea atroviridis*), *T*. *virens* strain IMI 206040 (teleomorph *Hypocrea virens*), and *T*. *reesei* strain QM6a (ATCC13631; teleomorph *Hypocrea jecorina*) were used in this study. The fungi were cultivated at 28Â°C on either complete medium (PDA, PDB) or minimal medium (MM, containing [g/l]: MgSO_4_â€‰Â·â€‰7H_2_O 1, KH_2_PO_4_ 10, (NH_4_)_2_SO_4_ 6, tri-sodium citrate 3, FeSO_4_â€‰Â·â€‰7H_2_O 0.005, ZnSO_4_â€‰Â·â€‰2H_2_O 0.0014, CoCl_2_â€‰Â·â€‰6H_2_O 0.002, MnSO_4_â€‰Â·â€‰6H_2_O 0.0017, glucose 10) on plates and in liquid culture, respectively. Plate confrontation assays were performed by cultivating *Trichoderma* together with *Rhizoctonia solani* on PDA plates covered with a cellophane membrane at 28Â°C. After direct contact between the two fungi, mycelium of *Trichoderma* was harvested from the confrontation zone. For RNA isolation, 30 mg fungal mycelium was grinded in liquid nitrogen and RNA isolated using the peqGOLD TriFast Solution (PeqLab, Erlangen, Germany) according to the manufacturerÂ´s instructions.

For cDNA synthesis the Revert Aid H Minus First Strand cDNA Synthesis Kit (Fermentas, Vilnius, Lithuania) was used according to the manufacturerÂ´s instructions with a combination of an oligo(dT)_18_ and a random hexamer primer. The sequences for the respective primer pairs for cDNA amplification of the reference gene *sar1* and the genes encoding the putative receptors of class VIII identified in the *Trichoderma* genomes are given in Additional file 3. Transcript quantification was performed with the following PCR program (initial denaturation for 120 s at 95Â°C, 50 cycles with 95Â°C for 20 s, 60Â°C for 20 s and 72Â°C for 20 s) on an Eppendorf (Hamburg, Germany) realplex2S Mastercycler using the IQ SYBR Green Supermix (Bio-Rad, Hercules, CA) and 25 Î¼l assays with standard MgCl2 concentration (3 mM) and with final primer concentrations of 100 nM each. All assays were carried out in 96-well plates covered with optical tape. PCR efficiency was determined from a single tube reaction set-up as described [74] and expression ratio was calculated according to Pfaffl [75]. All samples were analyzed in three independent experiments with three replicates in each run. Statistical analysis was done by relative expression analysis with REST software using the Pair Wise Fixed Reallocation Randomisation Test [76].

## Competing interests

The authors declare that they have no competing interests.

## Authors contributions

SZ conceived the study, drafted the manuscript, and performed in silico analyses together with MO. SG contributed to gene identifications and performed the cultivations and RT-qPCR experiments. All authors read and approved the final manuscript.

## Supplementary Material

Additional file 1 **Cladogram of the phylogenetic relationship of putative GPCRs of classes I to IX of*A.nidulans*and their*Trichoderma*orthologues.** The Figure shows the phylogenetic relationship of the newly identified putative GPCRs of classes I to IX of *T*. *atroviride*, *T*. *virens*, and *T*. *reesei* with their orthologues previously identified in *A*. *nidulans*[1]. The tree was generated using the CLUSTAL X alignment. Click here for file

Additional file 2 **PTH11-like GPCRs of*T.atroviride,T.virens*, and*T.reesei.*** The table gives the protein IDs of PTH11-like GPCRs identified in the genomes of the three *Trichoderma* species. The proteins are arranged according to the phylogenetic analysis (Figure 5). * Proteins containing a CFEM domain. Click here for file

Additional file 3 Primer pairs used for transcript quantification of class VIII members. Click here for file
