# Supplementary figures and images for: Comparative analysis of the repertoire of G protein-coupled receptors of three species of the fungal genus Trichoderma
Source: BMC Microbiol. 2013 May 16;13:108. doi: 10.1186/1471-2180-13-108 (PMC3664084; doi:10.1186/1471-2180-13-108)

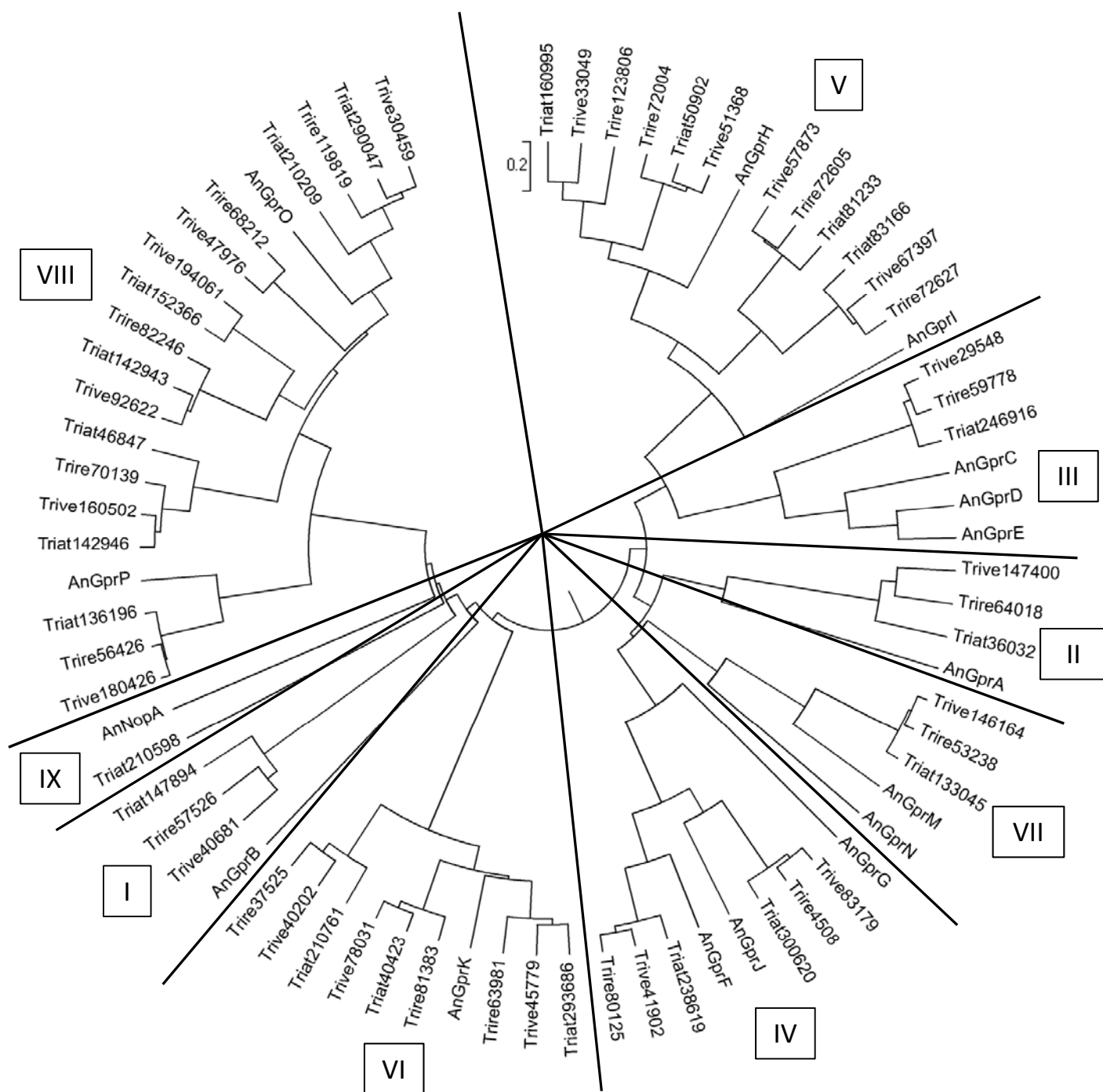

Supplement: Additional file 1 — Cladogram of the phylogenetic relationship of putative GPCRs of classes I to IX ofA.nidulansand theirTrichodermaorthologues. The Figure shows the phylogenetic relationship of the newly identified putative GPCRs of classes I to IX of T. atroviride, T. virens, and T. reesei with their orthologues previously identified in A. nidulans[1]. The tree was generated using the CLUSTAL X alignment. [file 1471-2180-13-108-S1.pdf]
