# Supplementary material for: Comparative analysis of the repertoire of G protein-coupled receptors of three species of the fungal genus Trichoderma
Source: BMC Microbiol. 2013 May 16;13:108. doi: 10.1186/1471-2180-13-108 (PMC3664084; doi:10.1186/1471-2180-13-108)
Supplement: Additional file 2 — PTH11-like GPCRs ofT.atroviride,T.virens, andT.reesei. The table gives the protein IDs of PTH11-like GPCRs identified in the genomes of the three Trichoderma species. The proteins are arranged according to the phylogenetic analysis (Figure 5). * Proteins containing a CFEM domain. [file 1471-2180-13-108-S2.pdf]

Additional File 2: PTH11-like GPCRs of *T. atroviride*, *T. virens*, and *T. reesei*. Proteins are arranged according to the phylogenetic analysis (Fig. 5). * Proteins containing a CFEM domain, # proteins in the clade specific for the two mycoparasitic species *T. atroviride* and *T. virens.*

T. atroviride	***T. virens***	***T. reesei***
ID 152180	ID 69487	ID 69500
ID 222553	ID 25302	
	ID 78159	ID 122795
ID 297464		
ID 7339	ID 57355	ID 45573
ID 91914	ID 143640	ID 5647
	ID 39587	
ID 300847*	ID 28615*	ID 27992*
	ID 63258*	ID 27983*
ID 30137*	ID 44825*	
ID 252553*	ID 51635*	ID 62462*
	ID53566	ID 53452
ID 316718	ID 42454	ID 66786
	ID 53608	
ID 320199	ID 221904	ID 106082
ID 322695	ID 204543	
ID 130989		ID 55561
ID 85568		
ID 88792		
ID 79664	ID 52498	ID 67334
ID 35699	ID 36530	ID 61354
	ID 47179	ID 122824
ID 158188	ID 379171	ID 40156
	ID 80528	
	ID 111686	ID 70967
ID 156014		ID 58767
		ID 41260
	ID 29230	ID 110744
ID256241	ID 112017	ID 111861
ID 46092	ID 152867	

ID 314211#	ID 140068#	
	ID 46790#	
ID 83320#	ID 53006#	
	ID 53253#	
	ID 44370#	
	ID 53542#	
	ID 51581#	
ID 34593#	ID 28393#	
ID 80140	ID 187849	ID 69904
ID 156579	ID 68995	
ID 46317	ID 52059	ID 41425
ID 132642	ID 58793	ID 105224
ID 86665	ID 78137	ID 57101
ID 79255	ID 73911	ID 124113
	ID 223762	
ID 306964	ID 70756	ID 110339
ID 44659		ID 66673
	ID 33081	
ID 322916	ID 71185	ID 76763
	ID 35501	ID 39587
	ID 15465	ID 121990
ID 319558	ID 200188	ID 109146
	ID 225906	
	ID 69879	
	ID 47845	
ID 153140	ID 213239	ID 78499
ID 134069		
ID 222777	ID 55967	ID 107042
ID 130873	ID 54271	ID 103694
ID 220212	ID49814	ID 82041
