# Supplementary material for: Comparative analysis of the repertoire of G protein-coupled receptors of three species of the fungal genus Trichoderma
Source: BMC Microbiol. 2013 May 16;13:108. doi: 10.1186/1471-2180-13-108 (PMC3664084; doi:10.1186/1471-2180-13-108)
Supplement: Additional file 3 — Primer pairs used for transcript quantification of class VIII members. [file 1471-2180-13-108-S3.pdf]

Additional File 3: Primer pairs used for transcript quantification of class VIII members.

gene	forward primer (5’ to 3’)	reverse primer (5’ to 3’)
Triat290047	CTCACCCTCATCCACAGACTC	CAAAGACCAGCACATCCGAC
Triat142946	TGAGCACGACGAACGAACC	CCATGCCAGAACTGTTCTACAG
Triat136196	CTACCAGCGGTTCGGATTTG	CGAGGGTCTCAAGCATGATG
Triat210209	CGTCTTCGCATTCTTTATTGGTGGC	TCGAACCCCTTGTAGCCGTAGATG
Triat142943	TCCCGAGGTGGCATCAAG	CCGAGAACTCCAATCATAGAC
Triat152366	CGATGATAGGTTCACTGGG	CGTATTTCTCGGGCTTTCG
Triat46847	GCACTGGGCGATTATCACATC	CCAGATGTCGAATTTGCCTG
Triat sar1	CTCGACAATGCCGGAAAGACCA	TTGCCAAGGATGACAAAGGGG
Trive30459	TTAGAACGCCCGCTTGGAGG	GCCAGCGAATCGCATAGAGAA
Trive47976	CATTACACCCACGCTTCATCTC	CGAACTTGTGAGGAACCCATCT
Trive160502	GAGTCCAGCGATACGGGTTTG	TATCAAATCTGCCGGGTGCC
Trive194061	TGCCGCCACCATCTTTCCATA	CCAATCGAAGGCATTTAGAATGC
Trive92622	CTTGCGTTCGTAGGGACTGG	GATGAGAAGAGCCGTAAATGTCG
Trive180426	GCGTCATCCTGCCGTGG	GATGATGCCGCCCAAGACAG
Trive sar1	GCGAACACCAGCAGAGCC	GTCACGACGATACCAACGAT
Trire119819	CTCATGTTCGTCAGCCTCGG	GATAGCTTCTCTCGGGCCAAC
Trire68212	CATTGGCGTGCGTGCTTCTAC	GAAATGTATCACGGCGGCAACC
Trire70139	GCCTCGTCATTCATACCCCTG	GCAGCAGAGCCGTCACATG
Trire82246	CTGGTGGGCATAGTCATCCTC	CGGTCCCTACAAACGCAAGTG
Trire56426	GTCGTAGCCGCCATCTTCTTCAT	GCCGACGAAGAAGCCGACT
Trire sar1	TGGATCGTCAACTGGTTCTACGA	GCATGTGTAGCAACGTGGTCTTT
